# Intestinal Probiotic Lysate Modified Bifunctional Nanoparticle for Efficient Colon Cancer Immunotherapy

**DOI:** 10.3390/pharmaceutics17020139

**Published:** 2025-01-21

**Authors:** Manfang Zhu, Hongkui Chen, Xiaohua Chen, Yueyang Zhang, Xiayu Chen, Bailing Zhou, Xingmei Duan, Na Zhou, Xin Zhang

**Affiliations:** 1 Department of Pharmacy, Personalized Drug Therapy Key Laboratory of Sichuan Province Sichuan Academy of Medical Sciences, Sichuan Provincial People’s Hospital, School of Medicine, University of Electronic Science and Technology of China, Chengdu 610072, China; zhumanfang2022@163.com (M.Z.); zyy0108291@163.com (Y.Z.); duanxingmei2003@163.com (X.D.); 2 School of Pharmacy, State Key Laboratory of Quality Research in Chinese Medicines, Laboratory for Drug Discovery from Natural Resources & Industrialization, Macau University of Science and Technology, Macau 999078, China; 3230007907@student.must.edu.mo; 3 State Key Laboratory of Biotherapy and Cancer Center, West China Hospital, Sichuan University, Chengdu 610041, China; cxh_an@163.com (X.C.); orange_xiayu621@163.com (X.C.); zhoubailing@scu.edu.cn (B.Z.)

**Keywords:** siRNA delivery, intestinal probiotic, immune therapy, CD47

## Abstract

**Background:** In cancer immunotherapy, gene therapy has become a promising strategy through the introduction of immunostimulatory components into its formulation. However, ideal non-viral gene delivery platforms capable of simultaneously maintaining a high delivery efficiency and immune activation are still in demand. As an intestinal probiotic, *Lactobacillus reuteri* has potential correlation with cancer progression. Its unique antigenicity also confers its immunomodulatory activity. **Method:** We engineered a new non-viral siRNA delivery system, DMPLAC. By wrapping the lysate of *Lactobacillus reuteri*, it is expected to enhance the anti-cancer immunostimulatory properties. **Result:** Supported by certain internalization pathways, the prepared DMPLAC nanoparticles showed high siRNA delivery efficiency in vitro (up to 97.62%). They also strongly promoted the maturation and activation of immune cells, including dendritic and T cells, both in vitro and in vivo. By loading siRNA targeting the immune checkpoint CD47 gene, the DMPLAC/siCD47 complex strongly suppressed the growth of multiple colon cancer models through local administration with high safety. **Conclusions:** Our study developed a novel intestinal probiotic lysate-based gene delivery system with dual immunomodulatory abilities, suggesting a potential strategy for cancer immunotherapy.

## 1. Introduction

Cancer is the second leading cause of death in the world, after cardiovascular disease [1]. According to the Global Cancer Statistics 2020, of all cancers, colorectal cancer is the third most common and the second deadliest worldwide [2]. Traditional methods for the treatment of colorectal cancer include surgical resection and chemotherapy. Gene therapy has recently emerged as a new method and has become a topic of research interest in the field of antitumor therapy [3,4]. siRNA-based gene therapy is one of the means of colorectal cancer therapy, and it plays a role in silencing anti-apoptosis or introducing related genes that promote apoptosis [5,6]. Although siRNA therapy in colorectal cancer has attracted much attention, its therapeutic effect still needs to be further improved.

Tumor immunotherapy is an antitumor approach based on activation of the immune response. Unlike treatment methods such as surgery and chemotherapy, the target of immunotherapy is not a tumor tissue but an immune system, indicating its potential for enhanced antitumor effect [7]. Gene therapy is the treatment of diseases based on replacing defective genes or adding new genes. Attributed to gene delivery, gene editing, and other functions, gene therapy could achieve therapeutic effects by delivering therapeutic nucleic acids [8]. The nanoskeleton-based non-viral gene vector, given its modifiability and physicochemical properties, can also play an immune-stimulating role. Therefore, based on non-viral gene therapy techniques, it would be helpful to develop new strategies for tumor immune gene therapy.

siRNA-based therapies are expected to play a key role in tumor treatment. One of the important aspects of siRNA preparation is the carrier, which protects the integrity of exogenous genetic material to achieve effective delivery [9,10]. An ideal in vivo delivery system can be an effective approach to ensure the treatment effect. Therefore, current research on tumor siRNA gene therapy has focused on further improving delivery efficiency, including developing new vectors, modifying the vector, and improving the targeting of the vector [11,12,13,14,15]. However, little research has been conducted to explore the therapeutic effects of the vector itself. As a matter of fact, nanoparticles themselves have high therapeutic potential because of their charged properties and skeleton modifiability. In immunogene therapy research, people are also looking for suitable immunostimulants and strategies.

Intestinal probiotics are a subset of gut microorganisms, and they exert a beneficial influence on immunotherapy for cancer [16,17,18,19]. *Lactobacillus reuteri* is an intestinal probiotic, which plays a positive role by promoting the proliferation of intestinal epithelial cells and producing lactic acid [20,21]. The peptidoglycan, extracellular polysaccharides, and other components of *Lactobacillus reuteri* are immunogenic [22,23]. Recently, it has been shown that bacteria can stimulate the production of anticancer cytokines and induce oxidative stress [24]. However, owing to the safety concerns of lipopolysaccharides and other ingredients of *Lactobacillus reuteri*, direct use of the bacteria for in vivo therapy is limited. It is not yet clear which component is responsible for the therapeutic effect, and the underlying therapeutic mechanism remains unclear. Considering that the bacterial lysate is a mixture of multiple components with different solubilities, suitable methods that can be introduced into existing nonviral vector skeletons must be developed.

Previously, we modified the amphiphilic polymer methoxypoly(ethylene glycol)poly(ε-caprolactone) (mPEG-PCL)with cationic lipid N-[1-(2,3-dioleoyloxy) propyl]-N,N,N-trimethylammonium methyl-sulfate (cationic lipid DOTAP, Avanti). DMP (DOTAP-mPEG-PCL) was prepared by DOTAP self-assembly with mPEG-PCL [25,26]. In many nucleic acid-related gene therapy studies, DMP has been successfully applied [27,28]. In this study, we encapsulated *Lactobacillus reuteri* lysates into the DMP skeleton to prepare a lysate-based gene-delivery vector DMPLAC. By loading siRNA targeting CD47, we speculated that the DMPLAC/siCD47 complex could disrupt immune suppression and enhance immune response. The gene delivery and immunostimulatory abilities of the DMPLAC vector were further studied, and the anticancer ability of the DMPLAC/CD47 complex in several mouse tumor models, such as subcutaneous and abdominal tumor models, was evaluated.

## 2. Materials and Methods

### 2.1. Cell Lines and Animals

CT26 and HEK293T cells were purchased from ATCC. Dulbecco’s modified Eagle’s Medium (DMEM) containing 10% Fetal Bovine Serum (FBS) was utilized (Thermo Fisher, Waltham, MA, USA). BALB/c mice were obtained from Beijing HFK Biotechnology Co., Ltd. (Beijing, China). All mice were maintained under specific pathogen-free conditions. All mouse experiments were conducted under the guidance of the Animal Ethics Committee of the General Bureau of Health Research of Sichuan University, in accordance with the guidelines for experimental animals of Sichuan University and standard operating procedures (SOPs).

### 2.2. Preparation and Characterization of Nanoparticles

The lysate of *Lactobacillus reuteri* was prepared as follows. We used a high-pressured viscolizer to break the wall of bacterial (1000 MPa, 60 min) [29], and then we filtered the suspension through a 0.22 μm filter membrane (Millipore, Darmstadt, Germany) to obtain the final lysate.

To prepare the DMPLAC, first, we took methylene chloride (Sigma-Aldrich, San Diego, CA, USA) and dissolved mPEG2K-PCL2K and DOTAP (1,2-dioleoyl-3-trimethylammonium propane) (90:10, *w*/*w*) (Xi’an ruixi Biological Technology, Xi’an, China) in it. To remove the dichloromethane, the mixture was poured into a rotary evaporator and evaporated for 45 min (130 RPM). Then, the solvent evaporated and formed a lipid film. The lytic solution of *Lactobacillus reuteri* was added to the beaker, and it self-assembled into micelles at 45 °C. DMP and DMPLAC’s size distribution and ζ potential were analyzed using a ZetaSizer Nano ZS instrument (Malvern Instruments, Ltd., Malvern, UK). Morphological analysis of the prepared particles was conducted using transmission electron microscopy (TEM). A gel delay assay was used to evaluate the siRNA-binding ability of DMPLAC nanoparticles.

### 2.3. Gram Faerbung Staining

First, the sample was fixed on a slide. The samples were dyed for 5 min with crystal violet and Gran’s iodine solution (Thermo Fisher, America). The complete staining of Lactobacillus reuteri and its lysates was observed and analyzed using a fluorescence microscope (Olympus, Tokyo, Japan).

### 2.4. Stimulation of BMDC

To prepare bone marrow-derived DCs (BMDCs), total bone marrow cells were collected from the femurs and tibias of Balb/c mice, and all cells were suspended in RPMI-1640 medium supplemented with 10% FBS. After the red blood cells ruptured, the remaining ones were cleaned with a full culture solution. Bone marrow cells were re-suspended in a complete culture medium supplemented with 10% fetal bovine serum (20 ng GM-CSF/mL). After 6 days, BMDCs were seeded in a six-well plate, and LPS (0.1 mg/mL), DMP, LAC (30 μg), or DMPLAC (30 μg LAC) was added. The cells were then cultured for another 48 h. To study the expression of markers of DC cell maturation (CD11c, CD80, CD86), cells were collected and flow cytometry (ACEA NovoCyte^TM^, San Diego, CA, USA) was used. All data were statistically analyzed using Prism 7 software (GraphPad Software, La Jolla, CA, USA) and one-way ANOVA.

### 2.5. Flow Cytometric Analysis of Activated T Cells In Vitro

In order to study the reactions of T cells and antigen-presenting cells (APCs) under the action of DMPLAC, we co-cultured DMPLAC with T cells or DC cells. First, we obtained mouse bone marrow cells, which were induced to differentiate into BMDCs under the action of GM-CSF (Abcam, Cambridge, UK). After six days, BMDCs were collected using a cell scraper and reseeded into a six-well plate at a 1:1 ratio (1.5 × 10^7^ cells per well). For T cells, cells from the lymph nodes were extracted and co-cultured with BMDCs (2.5 × 10^6^ cells/well). At the same time, LPS (Sigma-Aldrich, America) (0.1 mg/mL), DMP, LAC (30 μg), or DMPLAC (30 μg LAC) was added. The cells were left in the incubator for another 48 h. Then flow cytometry was used to detect T cell activation and cytotoxicity (CD3, CD4, CD69). All data were statistically analyzed by one-way ANOVA.

### 2.6. Immunostimulation of DMPLAC In Vivo

Next, the in vivo immune stimulation ability of DMPLAC system was studied. Briefly, BALB/c mice were intramuscularly injected with DMP, LAC, and DMPLAC. After the mice were kept for 48 h, they were killed, and lymphocytes from their lymph nodes and spleens were isolated. Lymphocytes were stained with CD80, CD86, CD11C, CD3, CD4, CD8, CD49b, and CD69 antibodies and analyzed using flow cytometry.

After the intraperitoneal model was established for TME, DMP, LAC, and DMPLAC were intraperitoneally injected for three days in a row. Tumor tissues were then separated using flow cytometry. All data were statistically analyzed by one-way ANOVA.

### 2.7. RNase-Protection Assay

In order to investigate whether DMPLAC can successfully protect siRNA in the presence of RNase, we conducted enzyme protection experiments. Naked siNC or DMPLAC/siNC was incubated with RNase A (Solarbio, Beijing, China). After treatment at 37 °C for different durations, sodium dodecyl sulfate (SDS, 0.24 mg/mL) was added to the mixed solution. The samples were then placed at 70 °C for 10 min. All samples were electrophoreted on 1% (*w*/*v*) agarose gel for 15 min at a voltage of 120 v.

### 2.8. Cell Viability Assay

In order to investigate whether the DMPLAC system was toxic to cells, we performed cytotoxicity tests. First, 293T and 3T3 cells were seeded into 96-well plates at a density of 3 × 10^3^ cells/well. After incubation in the incubator for 24 h, graded concentrations of DMPLAC, DMP, or PEI25K (a gold standard transfection agent) were added to the cells. At different timepoints (24 h, 36 h, and 48 h), we added MTT and incubated the plate under 37 °C for 4 h. Then DMSO was used to dissolve the reaction product, and absorbance was measured using an enzyme-labeled apparatus to calculate cell viability.

### 2.9. Hemolysis Assay

We collected blood from the eye sockets of mice and centrifuged it for 5 min. We removed the supernatant and added a phosphate buffer to the precipitate. The whole process was repeated 3–4 times until the supernatant ran clear. Then RBCs were diluted to 4% with phosphate buffer solution. The experiment was divided into a negative control group, a positive control group, a DMPLAC group, and a PEI25K group. Then, 500 μL of the 4% RBCs suspension was added to each group, and an equal volume of liquid was added (phosphate buffer solution was added to the negative control group, and Triton X-100 with a mass fraction of 0.5% was added to the positive control group. DMPLAC and PEI25K at concentrations of 50, 100, 200, 300 mg/mL were added, respectively). Then, the solutions were mixed evenly and incubated at 37 °C for 4 h. The samples were centrifuged at 2000 rpm for 5 min and photographed. The optical density of the supernatant was measured at a wavelength of 562 nm. The hemolysis rate was calculated as (sample absorbance—negative control absorbance)/(positive control absorbance—negative control absorbance) × 100. All data were statistically analyzed by *t*-tests.

### 2.10. In Vitro Transfection

CT26 cells were seeded into 24-well plates. Cells (3 × 10^4^) and 500 μL of the medium were added to each well (DMEM containing 10% FBS). After 24 h, the DMEM containing 10% FBS in each well was replaced with serum-free DMEM. Next, we mixed 1 μg of Cyanine 3–labeled siRNA and DMPLAC. Similarly, we mixed 1 μg siRNA, DMP, or PEI25K. Fifteen minutes after mixing the genes and vectors, the complexes were added to the cells. Four hours later, DMEM was replaced with a medium containing 10% FBS, and the plates were incubated at 37 °C. After 24 h, the cells were examined under a microscope and photographed, and transfection efficiency was measured by flow cytometry. All data were statistically analyzed by one-way ANOVA.

### 2.11. Endosomal Escape

DMPLAC nanoparticles of matching formulations as those used for the transfection experiments were used to deliver nucleic acid cargo labeled with FAM to enable the visualization of uptake. After incubating the DMPLAC/siRNA and CT26 cells or 4T1 cells for 2, 6, and 24 h, lysosomes were labeled with LysoTracker Red (Beyotime, Shanghai, China), cytomembranes were labeled with DIO (Beyotime, China), and cell nuclei were labeled with Hoechst (Beyotime, China). Endosomal escape was visualized using a confocal microscope (Nikon AXR, Tokyo, Japan).

### 2.12. Trafficking Mechanism Analysis

In order to investigate the cellular internalization mechanism of DMPLAC, we used several inhibitors of cellular uptake pathways to explore it. Different inhibitors represent different signal transduction pathways. And the endocytosis pathway and intracellular transport mechanism of DMPLAC were investigated. CT26 cells with a 3 × 10^4^ cell/pore density were inoculated on a 24-well plate. After incubation for 24 h, the cells were pre-incubated with the different inhibitors for 1 h. The medium was discarded, and 1 μg cyanine 3-siRNA was transfected with DMPLAC. After 24 h, the transfection efficiency of DMPLAC was measured by flow cytometry. Different endocytosis inhibitors included methyl-β-cyclodextrin (M-β-CD) (10 mM), genistein (30 mM), CPZ (0.25 mg/mL), filipin III (1 mg/mL), and amiloride (100 mM). For fluorescence microscopy, CT26 cells were seeded in chamber slides (Millicell, 4-well glass) and treated with different inhibitors, in accordance with the appropriate protocols. After transfection for 24 h, nuclei were stained with Hoechst (1 mg/mL, Solarbio), and plasma membranes were stained with Dil (10 mg/mL, Beyotime). CLSM (Zeiss, Oberkochen, Germany) was used to analyze the different inhibitors’ fluorescence levels. All data were statistically analyzed by one-way ANOVA.

### 2.13. Quantitative Real-Time PCR (RT-PCR)

Down-regulated expression of CD47 in CT26 cells was detected by RT-PCR. Briefly, CT26 cells were seeded at a density of 3 × 10^4^ cells/well in 24-well plates. Next, 48 h after transfection with DMPLAC, DMPLAC/siNC, DMP/siCD47, PEI25K/siCD47, or DMPLAC/siCD47 (2 μg siCD47), the total RNA was harvested from the cells. The expression level of CD47 was detected using an SYBR Green ER quantitative PCR SuperMix universal kit (Vazyme). mRNA was converted to cDNA using SuperScript II (Vazyme). The primers were CD47: forward 5′-TGGCTATACTCCTGTTCTGGG-3′, reverse 5′-TTACAATGAGGCCAAGTCCAG-3′. CT26 amplification was used as an internal control. All data were statistically analyzed by one-way ANOVA.

### 2.14. Western Blot Analysis

CT26 cells were seeded in six-well plates for 24 h and treated with phosphate buffer solution, DMPLAC, DMPLAC/siNC, DMP/siCD47, PEI25K/siCD47, or DMPLAC/siCD47 (4 μg siCD47) for 48 h. After transfection, the cells in each group were washed and lysed with RIPA lysis buffer supplemented with protease inhibitors at 4 °C for 20 min. Then, the homogenate was collected, and the protein concentration was determined by Bradford method. Total protein lysates were separated by SDS-PAGE and transferred to PVDF membranes. The membrane was incubated with primary antibodies against CD47 (1:5000 dilution; Abcam) overnight. Then, incubation was continued with HRP-conjugated secondary antibody for 1 h. β-actin (1:5000 dilution; Abcam) was used as the loading control. The density of the target protein signals was visualized using an eBlot Touch Imager. Quantitative analyses were performed using ImageJ software v1.54m.

### 2.15. Expression of CD47 on Cells

To verify the reduction in CD47 in CT26 cells after transfection, we stained the cells with anti-CD47 and performed flow cytometry. Briefly, CT26 cells were seeded in 24-well plates and treated with DMPLAC, DMPLAC/siNC, DMP/siCD47, PEI25K/siCD47, or DMPLAC/siCD47 (1 μg siCD47) twice for three days. On day 3, the cells were collected and incubated with anti-CD47 antibody for detection. All data were statistically analyzed by one-way ANOVA.

### 2.16. Animal Models

On day 0 of the experiment, female BALB/c mice (5 weeks old) were subcutaneously inoculated with 2 × 10^6^ CT26 cells. Tumor size was measured every alternate day, and tumor volume was calculated using the following equation: V = (length × width^2^)/2. Body weight was monitored daily. To demonstrate the anti-tumor efficiency of DMPLAC, on day 3, tumor-bearing mice were randomly separated into four groups that received 10 intratumoral injections of normal saline, DMP, LAC, or DMPLAC (100 μL). On day 13, the animals were euthanized and the tumors were removed for photography.

To test the efficacy of DMPLAC/siCD47, after grouping on day 3, DMPLAC, DMPLAC/siNC, or DMPLAC/siCD47 was used (10 μg siRNA per mouse or a corresponding dose of DMPLAC to 10 μg siRNA). On day 15, the animals were euthanized and the tumors and main organs were removed for further analysis. All tumors were weighed and processed for histopathological analysis.

For the intraperitoneal model, CT26 cells (2.5 × 10^5^ cells) were intraperitoneally injected into female Balb/c mice. On day 3, mice were randomly divided into four groups and intraperitoneally injected with normal saline (NS), DMPLAC, DMPLAC/siNC (10 μg siNC), or DMPLAC/siCD47 (10 μg siCD47). The body weights of the mice were monitored daily. On day 12, the mice were euthanized and their tumors were photographed and weighed. The weight change curve and tumor volume change curve data of the mice were analyzed by two-way ANOVA, and the remaining data were analyzed by one-way ANOVA.

### 2.17. Immunohistochemical Analysis

Histological specimens of formalin-fixed paraffin-embedded tissue sections were deparaffinized and rehydrated. The tissues were stained with CD8 (1:250, Abcam), IFN-γ (1:500, Abcam), CD31 (1:50, Abcam), TNF-α (1:200, Abcam), and CD47 (1:50, Abcam) to analyze the infiltrating immune cells in the model. To detect apoptosis, a TUNEL kit (Promega) was used to stain paraffin sections.

### 2.18. Tissue Dissociation and Flow Cytometry

Tumor samples were minced with scissors and incubated with 1 mg/mL collagenase IV in RPMI-1640 medium for 30 min at 37 °C. Single-cell suspensions were prepared by filtering through a 70 μm nylon filter strainer. The cells were stained with appropriate dilutions of various combinations of the following fluorochrome-conjugated antibodies: anti-CD86, anti-CD4, anti-CD11b, anti-F4/80, anti-CD3, anti-CD4, anti-CD8, anti-CD69, anti-80, anti-CD11C, and CD25. All flow cytometry data were acquired and analyzed as described above.

### 2.19. Routine Blood and Serum Biochemical Examinations

Normal saline and DMPLAC/siCD47 were intravenously injected into female Balb/c mice, and blood was collected after 24 h. Mean corpuscular volume (MCV), mean platelet volume (MPV), hemoglobin (HGB), mean corpuscular hemoglobin concentration (MCHC), red blood cells (RBCs), platelet distribution width (PDW), mean corpuscular hemoglobin (MCH), red blood cell distribution width (RDW), and hematocrit (HCT) were assessed. To assess liver and kidney function, we analyzed the levels of alanine aminotransferase (ALT), aspartate aminotransferase (AST), uric acid (UA), and total protein (TP).

### 2.20. Statistical Analysis

Data analysis was performed using *t* tests and one- and two-way analyses of variance (ANOVA). All data are presented as mean ± standard deviation (SD).

## 3. Results

### 3.1. Preparation and Characterization of the DMPLAC/siRNA Complex

**Figure 1:**
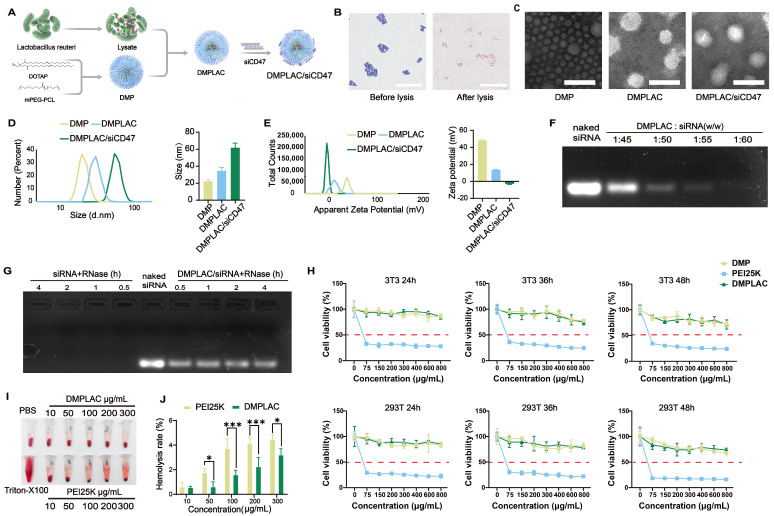
Synthesis and physicochemical property of DMPLAC. (**A**) Synthesis process of DMPLAC/siCD47. (**B**) Before and after Lactobacillus reuteri lysis (scale bar: 10 μm). (**C**) Electron microscope image of DMPLAC (scale bar: 100 nm). (**D**,**E**) Size and Zeta potential of DMPLAC. (**F**) Gel block electrophoresis of siRNA by DMPLAC. (**G**) Electrophoresis analysis: DMPLAC protects siRNA from RNase. (**H**) Toxicity of PEI25K, DMP, and DMPLAC in 293T cells. (**I**) Hemolysis ratio of PEI25K and DMPLAC. (**J**) Qualitative analysis of hemolysis. (* *p* < 0.05, *** *p* < 0.001) (Data were analyzed by *t*-test).

DMPLAC was prepared by embedding the lysate of *Lactobacillus reuteri* in a DMP nanoparticle framework (Figure 1A). First, we cultured the bacteria for 16 h and collected them to obtain *Lactobacillus reuteri* lysates through a series of high-pressure wall-breaking, ultrasonic, and membrane filtration procedures. The bacteria and lysates were stained using the Gram staining method. Before lysis, *Lactobacillus reuteri* was stained purple because it is a gram-positive bacterium with a thick cell wall. The lysate stained red, indicating that the bacteria were effectively lysed (Figure 1B). In this study, DMP was prepared by self-assembly using DOTAP and mPEG-PCL polymers, and DMPLAC nanoparticles were prepared by wrapping the bacterial lysate in DMP. TEM analysis revealed that both DMP and DMPLAC were circular, and the diameter of DMPLAC increased (Figure 1C). The Malvern particle size analyzer was used to determine the particle size and zeta potential of the nanoparticles. As shown in Figure 1D,E, the size of DMPLAC increased, and the potential decreased. Next, we investigated the ability of DMPLAC to bind to siRNAs. As shown in Figure 1F, siRNA migration was completely inhibited when the siRNA/DMPLAC ratio was 1:60. Therefore, the ratio of 1:60 was chosen for this study. As shown in Figure 1G, naked siRNA was rapidly destroyed by RNase, according to the RNase-protection studies. However, the DMPLAC/siRNA complex did not degrade significantly, and bands were still visible after 4 h of treatment, indicating that DMPLAC protected the siRNA from degradation. Therefore, based on the above findings, we successfully prepared DMPLAC nanoparticles capable of delivering siRNAs. In addition, in 293T and 3T3 cells, the IC_50_ value of PEI25K was lower than 75 μg/mL, whereas the IC_50_ values of DMPLAC and DMP were both higher than 800 μg/mL, indicating the safety of the DMPLAC particles (Figure 1H). These results show that the DMPLAC nanoparticles can be used for injection. For the hemolysis assay, Triton-X100, which causes significant hemolysis, was used as a positive control. As shown in Figure 1I,J, DMPLAC solutions at concentrations of 300, 200, 100, 50, and 10 μg/mL did not cause obvious hemolysis; that is, after incubation with each DMPLAC solution, the hemolysis rate of RBCs was lower than 4%. In contrast, hemolysis was observed in the PEI25K group.

### 3.2. In Vitro Transfection and Cellular Uptake Mechanism of the DMPLAC/siRNA Complex

**Figure 2:**
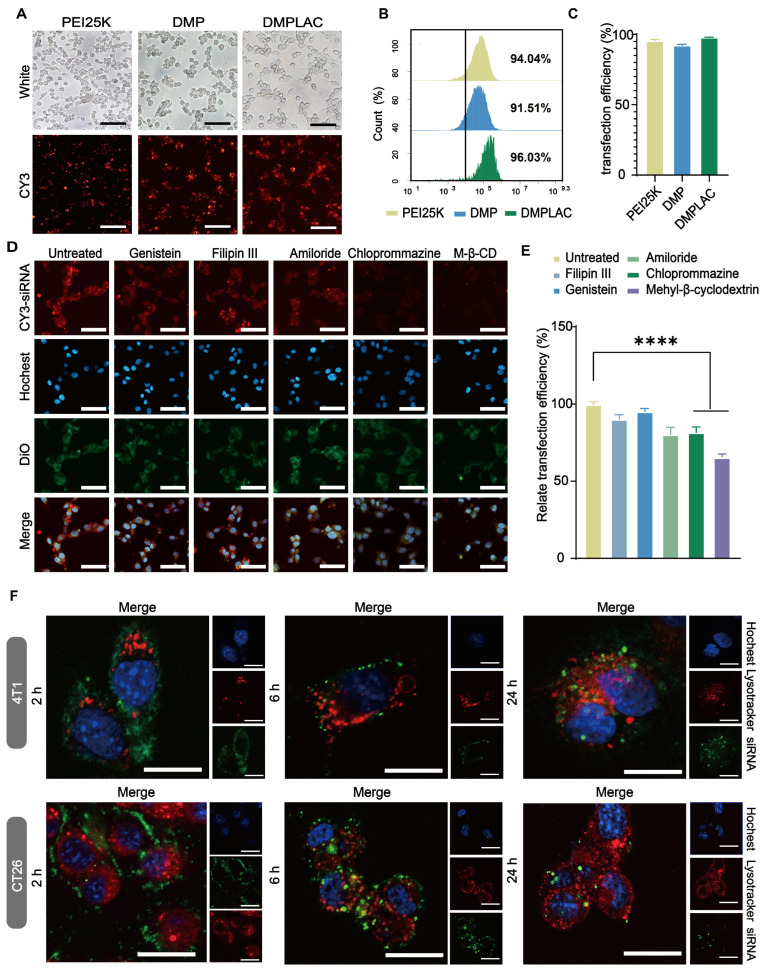
Transfection ability of DMPLAC in vitro. (**A**) Fluorescence transfection efficiency of CT26 cells after DMP and DMPLAC transfection (scale bar: 100 μm). (**B**,**C**) Transfection flow diagram and column diagram of DMPLAC/siRNA (*n* = 3). (**D**) DMPLAC/siRNA cell uptake mechanism map ((scale bar: 50 μm). (**E**) Analysis of DMPLAC/siRNA cell uptake mechanism (*n* = 3) (**** *p* < 0.0001). (**F**) Lysosomal uptake capacity of DMPLAC/siRNA in 4T1 cells and CT26 cells (scale bar: 50 μm). (All the above data were analyzed by one-way ANOVA).

To examine the siRNA delivery ability of DMPLAC, we performed transfection experiments on CT26 cells. Under fluorescence microscopy, we found that the delivery efficiency of the DMPLAC/CY3-siRNA complex was higher than that of PEI25K or DMP (Figure 2A). Flow cytometry analysis showed that the transfection efficiency of DMPLAC/siRNA was 96.03%, DMP was 91.51%, and PEI25K, the gold-standard transfection agent was 94.04% (Figure 2B,C). The gating strategies of the study regarding the transfection efficiency are shown in Figure S4. These results suggest that DMPLAC can be a novel nucleic acid delivery vector.

According to previous literature, whether the nanoparticle can successfully fulfill its delivery functions depends on whether it can help nucleic acids escape from lysosomes. Therefore, we explored the lysosomal escape ability of the DMPLAC/siRNA. As shown in Figure 2F, we found that, in both CT26 and 4T1, the red lysosome and FAM-siRNA had no evident colocalization, indicating that the DMPLAC/siRNA complex effectively prevented interaction with intracellular acidic lysosomes.

Owing to the excellent transfection efficiency of DMPLAC, we further investigated the cellular uptake mechanism of DMPLAC nanoparticles. Clathrin-mediated, caveolin-mediated, lipid raft–mediated, and macroendocytosis are the four basic processes involved in nanocarrier phagocytosis. After pretreatment of the cells with several inhibitors of internalization mechanisms (filipin III and genistein for caveolin-mediated endocytosis, amiloride for micropinocytosis, chlorpromazine for clathrin-mediated endocytosis, methyl-β-cyclodextrin for lipid raft–mediated endocytosis), as shown in Figure 2D, the red fluorescence in the methyl-β-cyclodextrin, chlorpromazine, and amiloride groups was weaker than that in the untreated group. In addition, the flow cytometry results (Figure 2E) demonstrated that methyl-β-cyclodextrin and chlorpromazine had great effects on cell uptake efficiency. Therefore, our findings suggested that endocytosis mediated by lipid rafts and clathrin played main roles in internalization.

### 3.3. Immune Stimulation Ability of DMPLAC

**Figure 3:**
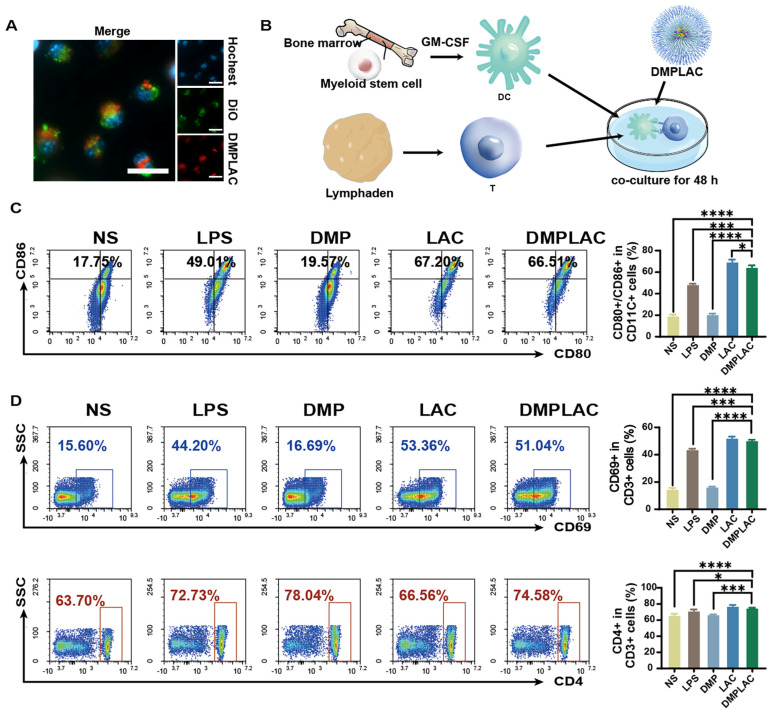
The ability of DMPLAC to activate immunity in vitro. (**A**) Fluorescence image of DMPLAC taken by BMDC (scale bar: 20 μm). (**B**) Schematic view of the experimental design. (**C**) Flow cytometry analysis of BMDCs stimulated by DMPLAC in vitro. Statistics of maturation and differentiation in BMDCs (* *p* < 0.05, *** *p* < 0.001, **** *p* < 0.0001) (*n* = 3). (**D**) Differentiation statistics of T cells by flow cytometry analysis (* *p* < 0.05, *** *p* < 0.001, **** *p* < 0.0001) (*n* = 3). (All the above data were analyzed by one-way ANOVA.).

Next, we investigated whether DMPLAC nanoparticles could effectively stimulate the immune response. First, we investigated whether DMPLAC could combine with DC in vitro. We isolated BMDCs from mice, labeled them with DMPLAC nanoparticles and rhodamine, and treated them with DMPLAC–rhodamine for 24 h. As shown in Figure 3A, we found that DMPLAC (red signal) colocalized with the cytomembrane (green signal), indicating that DMPLAC can effectively bind to DCs. The experimental design is shown in Figure 3B. Flow cytometry was performed for further analysis, the gating strategies are shown in Figure S1. As shown in Figure 3C, the DC maturation rate of the DMP group was 19.57%, whereas those of the LAC and DMPLAC groups reached 67.2% and 66.51%, respectively, indicating that *Lactobacillus reuteri* lysate as an antigen could effectively enhance the immunostimulatory ability of DMP nanoparticles. Next, we evaluated T-cell stimulation according to Figure 3D. Compared with the control and DMP groups, participation of the bacterial lysate significantly upregulated the expression of the activation-associated surface marker molecule CD69 (Figure 3D). The increase in the CD4+ ratio also indicated that DMPLAC enhanced T cell function after antigen presentation.

**Figure 4:**
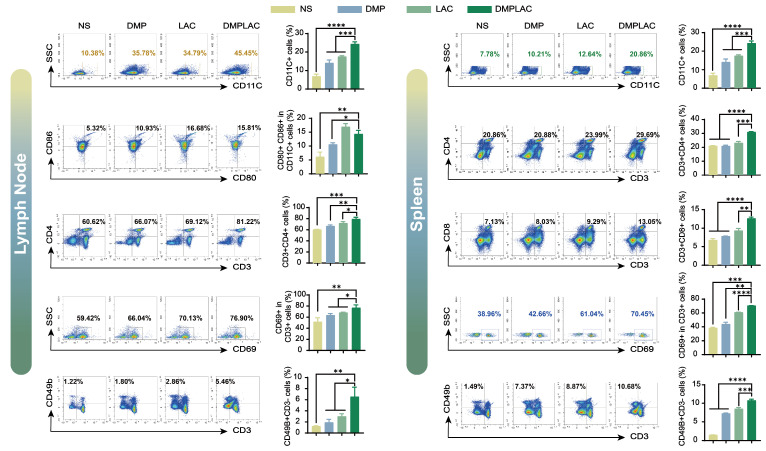
Immune stimulation of lymphocytes in lymph nodes and spleen after DMPLAC treatment (* *p* < 0.05, ** *p* < 0.01, *** *p* < 0.001, **** *p* < 0.0001) (*n* = 3). (All the above data were analyzed by one-way ANOVA.).

To further verify the immunostimulatory effect, we intramuscularly injected nanoparticles. The lymphocytes were then further analyzed by flow cytometry, and the gating strategies are shown in Figure S2. As shown in Figure 4, lymphocytes expressing CD11C, CD80, and CD86 in the DMPLAC group exhibited pronounced gains, demonstrating the ability of DMPLAC to induce DC maturation. The results showed that DMPLAC stimulated the activation of T cells, promoting the expression of CD4 and CD69. In addition, we evaluated changes in NK cells. The experimental data detected an increase in NK cells, suggesting that DMPLAC is an effective inducer of NK cell mobilization. Similar effects were observed in the spleen. According to the results for CD11C, DMPLAC showed the highest efficiency in stimulating the immune response, enabling proliferation of antigen-presenting cells. The increase in CD69, CD4+, and CD8+ T cells indicated the strengthening of immune function and cytotoxicity of T cells. Additionally, the NK cells of DMPLAC exhibited a greater increase.

**Figure 5:**
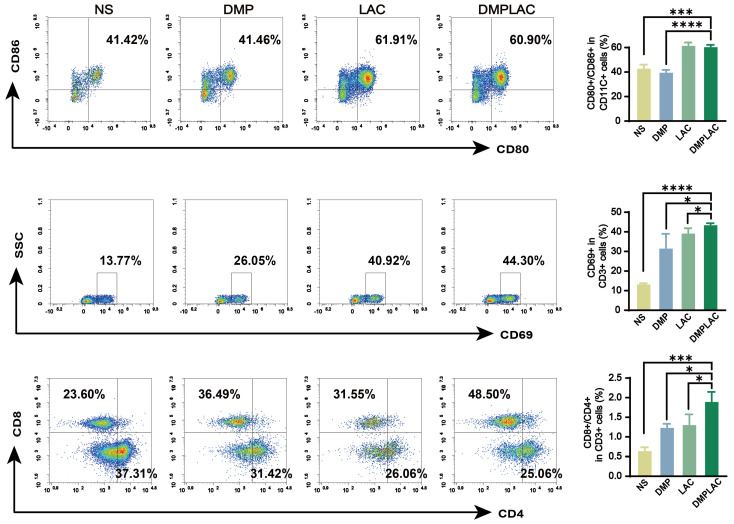
DMPLAC can effectively stimulate immunity in the body. Analysis of expression of markers of DC cells and T cells (* *p* < 0.05, *** *p* < 0.001, **** *p* < 0.0001) (*n* = 3). (All the above data were analyzed by one-way ANOVA.).

To further verify the role of DMPLAC in systemic immune stimulation, we treated the mice from the abdominal cavity metastatic model with DMP, LAC, and DMPLAC. The tumor tissues were then dissociated, and immune cell infiltration was detected. The gating strategies of the flow experiment regarding immunostimulation are shown in Figure S3. As shown in Figure 5, the proportion of mature DC in DMPLAC group was high, reaching 60.9%, suggesting that LAC could efficiently improve DCs’ maturation and the ability to present antigens. We also found that T cells were relatively activated in the DMP, LAC, and DMPLAC groups, as evidenced by upregulated expression of the early activation marker CD69 [30]. Moreover, compared with the control group, the CD8+/CD4+ ratios in the DMP, LAC, and DMPLAC groups showed an increasing trend. Compared with the normal saline (NS) group, the percentage of CD8+ T cells in the DMP and LAC groups increased, and the percentage of CD8+ T cells in the DMPLAC group reached 48.5%, indicating that the injection of DMPLAC had the greatest potential to induce cytotoxic T cells.

Based on the experimental results presented above, we noted that DMP nanoparticles can act as gene vectors and adjuvants and can be enhanced using a bacterial lysate. In summary, DMPLAC effectively induced the activation and polarization of DCs and Mφ and triggered subsequent T cell responses.

### 3.4. Knockdown of CD47 In Vitro

**Figure 6:**
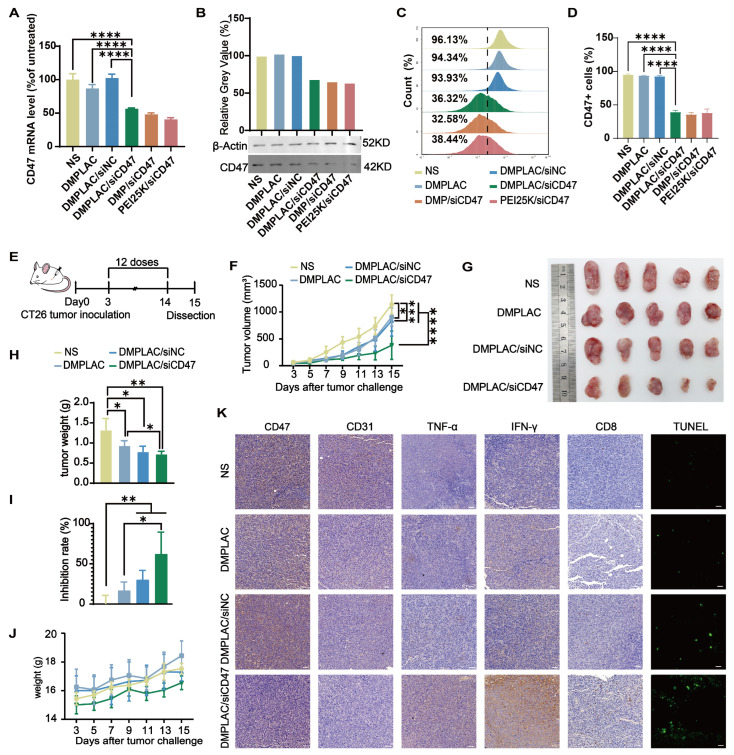
The DMPLAC/siCD47 complex knocks down the expression of CD47. (**A**) Level of CD47 mRNA in CT26 cells after DMPLAC/siCD47 treatment for 48 h (**** *p* < 0.0001) (*n* = 3). (Data were analyzed by one-way ANOVA). (**B**) Western blot analysis of CD47 protein level in CT26 cells after 48 h. (**C**) Flow cytometry analysis of CD47 expression. (**D**) Expression analysis of CD47 (**** *p* < 0.0001) (*n* = 3). (Data were analyzed by one-way ANOVA). (**E**) Schematic illustration of subcutaneous model design. (**F**) Average tumor growth curves in all treatment groups (* *p* < 0.05, *** *p* < 0.001, **** *p* < 0.0001) (*n* = 5). (Data were analyzed by two-way analysis). (**G**) Images of tumors were collected from each mouse in the NS group and each treatment group (*n* = 5). (**H**) Average mouse tumor weight in all treatment groups (* *p* < 0.05, ** *p* < 0.01) (*n* = 5). (Data were analyzed by one-way ANOVA). (**I**) Tumor inhibition rate in all therapeutic groups (* *p* < 0.05, ** *p* < 0.01). (Data were analyzed by one-way ANOVA). (**J**) Changes in body weight growth in mice in all therapeutic groups (*n* = 5). (Data were analyzed by two-way analysis). (**K**) Immunohistochemical evaluation and TUNEL analysis (scale bar: 50 μm).

Previous studies have reported that a key mechanism of immune escape from tumors is the overexpression of the immunosuppressive signaling molecule CD47. Therefore, we conducted in vitro experiments to verify the gene knockdown ability of the DMPLAC/siCD47 complex. First, we measured the level of CD47 mRNA in CT26 cells using QPCR. As shown in Figure 6A, the amount of CD47 mRNA was significantly reduced in the DMPLAC/siCD47 group compared with that in the other groups (*p* < 0.001). As shown by Western blotting (Figure 6B), the protein level of CD47 in the DMPLAC/siCD47 group was notably reduced, indicating that DMPLAC successfully and efficiently delivered CD47 siRNA into tumor cells and enabled it to exert its function. We then analyzed changes in CD47 expression using flow cytometry. The gating strategies of the study of expression of CD47 on CT26 are shown in Figure S5. As shown in Figure 6C,D, after transfection with DMPLAC/siCD47, the amount of CD47 on the membrane was reduced to 60.79%. The protein expression levels were further analyzed. According to these findings, the DMPLAC/siCD47 complex effectively inhibits the production of CD47 in CT26 cells via gene silencing.

### 3.5. The DMPLAC/siCD47 Complex Effectively Suppresses Colon Cancer

To confirm the therapeutic effect of DMPLAC/siCD47 complex, we established a subcutaneous tumor model. We established a subcutaneous tumor model to confirm the therapeutic effect of the DMPLAC/siCD47 complex (Figure 6E). The weights and tumor volumes of the mice were monitored during treatment. As shown in Figure 6F, the tumor volumes in the mice from the DMPLAC and DMPLAC/siNC groups were lower than those in the mice from NS and DMPLAC/siCD47 groups, and increased most slowly. Our results showed that the DMPLAC/siCD47 complex had the strongest antitumor effect, as the DMPLAC/siCD47 group had smaller tumors than the other three groups (Figure 6G). As shown in Figure 6H, the mean tumor weight of the DMPLAC/siCD47 group was 0.731 g. It was lower than those of the DMPLAC (0.94 g), DMPLAC/siNC (0.787 g), and normal saline (NS) (1.37 g) groups, indicating a remarkable inhibitory effect (Figure 6I). Immunohistochemical (IHC) and TUNEL experiments were used to analyze the therapeutic mechanism of the DMPLAC/siCD47 complex. As shown in Figure 6K, DMPLAC/siCD47 efficiently reduced the expression of CD47 in the tumor tissues. DMPLAC nanoparticles and CD47-gene silencing both effectively reduced tumor vessels, as shown by CD31 staining. In addition, an activated immune response was demonstrated using TNF-α and IFN-γ staining. TUNEL staining showed that DMPLAC promoted necrosis of the tumor tissue, and CD47 knockdown further enhanced this effect. In addition, H&E staining (Figure 8A) revealed no significant pathological changes in major organs treated with the DMPLAC/siCD47 complex. Additionally, as shown in Figure 6J, the body weight showed no obvious change, indicating the safety of DMPLAC/siCD47.

**Figure 7:**
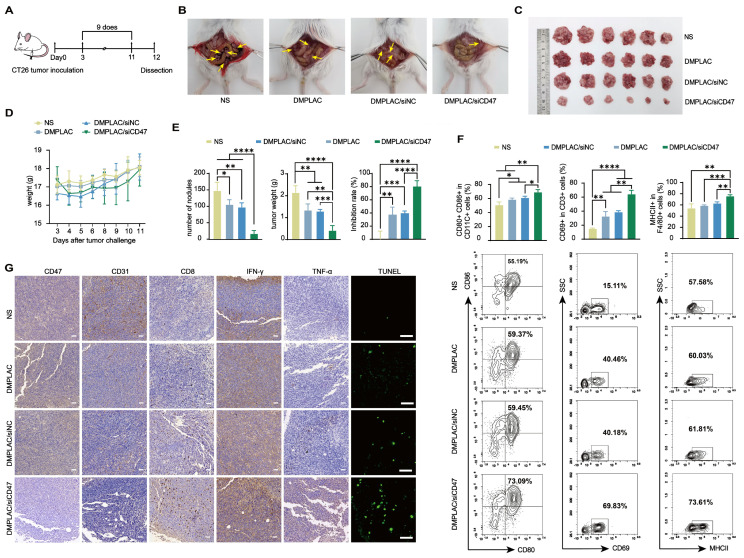
CT26 abdominal metastasis model for exploring the therapeutic effects of the DMPLAC/siCD47 complex. (**A**) Experimental design diagram. (**B**) Representative images of mice in the NS group and each therapeutic group. (**C**) Tumor nodule images taken from mice in the NS group and each therapeutic group (*n* = 6). (**D**) Changes in body weight in the NS group and each therapeutic group (*n* = 6). (Data were analyzed by two-way ANOVA). (**E**) The average number of tumor nodules collected in the NS group and each treatment group; the average tumor weights and Inhibition rate tumor weight in all therapeutic groups (* *p* < 0.05, ** *p* < 0.01, *** *p* < 0.001, **** *p* < 0.0001) (*n* = 6). (Data were analyzed by one-way ANOVA). (**F**) Immune activation and subsequent immune responses in TME during the experiment (* *p* < 0.05, ** *p* < 0.01, *** *p* < 0.001, **** *p* < 0.0001). (Data were analyzed by one-way ANOVA). (**G**) Immunohistochemical evaluation of tumor tissues (scale bar: 50 μm); TUNEL analysis of tumor tissues (scale bar: 50 μm).

**Figure 8:**
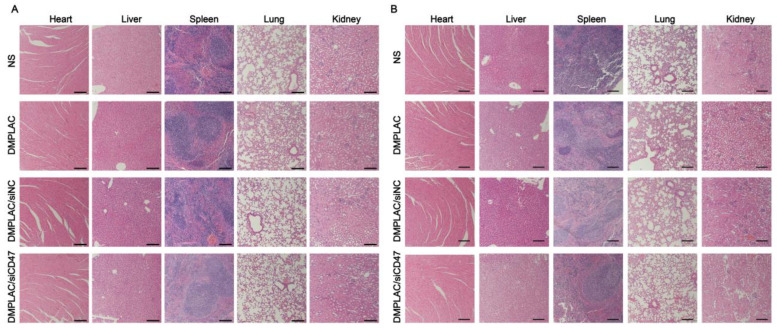
Immunohistochemical analysis of main organs. (**A**,**B**) Immunohistochemical analysis of main organs in the mouse model (scale bar: 100 μm).

In the abdominal model, we investigated the therapeutic potential of intraperitoneal injection of DMPLAC/siCD47 complex (Figure 7A). Enlarged abdominal images of the mice showed that both DMPLAC and DMPLAC/siNC improved abdominal symptoms associated with CT26 tumors, and the DMPLAC/siCD47 complex showed the greatest therapeutic effect (Figure 7B). After extracting the tumor tissue, it can be clearly seen that the DMPLAC/siCD47 treatment group had the fewest abdominal tumor nodules and the fewest pathological changes (Figure 7C). Additionally, as shown in Figure 7E, the weight of the tumors and the number of tumor nodes showed a decreasing trend, revealing the synergistic effect of DMPLAC and CD47-gene silencing in inhibiting tumor development. To understand how DMPLAC/siCD47 works, tumor tissues were collected for the evaluation of DC, T cell, and macrophage activation (Figure 7F). The gating strategies of the study regarding the TME of mice using an abdominal metastasis model are shown in Figure S6. Compared with normal saline (NS), the expression of co-stimulatory molecules CD80 and CD86 was significantly upregulated in the mice immunized with the complex containing DMPLAC, underscoring the importance of DMPLAC for successful antigen presentation and DC maturation, and this kind of maturation was enhanced by the participation of siCD47. The groups treated with DMPLAC, DMPLAC/siNC, and DMPLAC/siCD47 also showed higher levels of CD69+ T cells, which is representative of activation. Moreover, the CD47-mediated immune pathways were investigated. The promotion of MHC II + macrophages, representing M1-type macrophages, demonstrated that CD47-gene silencing was able induced proinflammatory effects during tumor cleaning. Subsequently, we studied the interior of the tumor tissue using IHC and TUNEL assays (Figure 7G). These results indicate that intraperitoneal administration effectively promoted the immune response and apoptosis of tumor cells to inhibit tumor development. DMPLAC/siCD47 increased the levels of the immune factors TNF-α and IFN-γ, and inhibited the production of CD47. The curves of body weight and HE analysis show that there were no significant pathological changes in the major organs (Figure 7D and Figure 8B).

The in vivo safety of the DMPLAC/siRNA system was evaluated using blood biochemistry and routine blood tests. According to our results, there were no significant changes or toxicity in the indicators ALT, AST, UA, TP, MCV, MPV, HGB, MCHC, RBCs, PDW, MCH, RDW, or HCT (Figure S7). This finding suggests that the DMPLAC/siCD47 complex has a high safety profile.

### 3.6. Anti-Tumor Activity of DMPLAC

We further evaluated the antitumor effect of DMPLAC in a subcutaneous model (Figure S8A). The tumor growth curves presented reflected a slower tumor growth rate in the DMPLAC group (Figure S8B). The dissected tumors depicted in Figure S8D showed a better tumor growth-inhibitory effect in the DMPLAC-treated group. In addition, as shown in Figure S8E,F, the average tumor weight in the DMPLAC group was 0.52 g, which was significantly lower than that in NS group and DMP group. Mouse weight (Figure S8C) suggested no apparent toxicity after administration.

## 4. Discussion

**Scheme 1:**
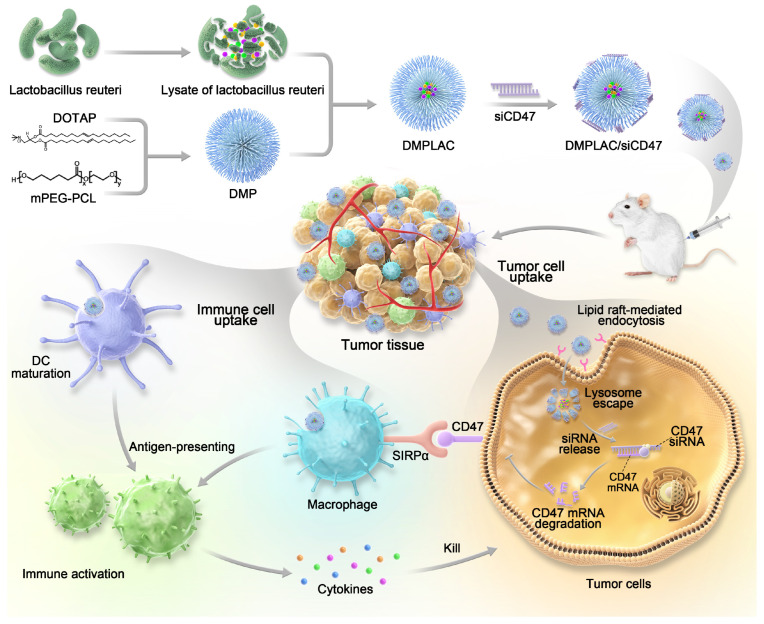
Synthesis and treatment of DMPLAC/siCD47 complex.

In this study, a multifunctional nanoparticle, DMPLAC/siCD47, was developed for colorectal cancer treatment by activating the immune response and disrupting immunosuppression. Our experimental results revealed that DMPLAC is an efficient immunostimulant that can deliver siRNAs into cells. Thus, the DMPLAC vector provides a novel strategy for activation of the immune response and an ideal vector for CD47 silencing. This synergistic treatment showed obvious antitumor efficacy in subcutaneous tumors and abdominal metastasis models. In summary, this study demonstrates the feasibility of combining TME regulation and immune checkpoint inhibitors (Scheme 1).

Immune stimulants can regulate the immune response to clear tumor cells. In order to improve the efficacy of gene therapy, immunotherapy is considered to be added to gene therapy. Finding a suitable method for combining gene therapy and immunotherapy has gradually become a hot research topic. In order to achieve this purpose, Dharmapuri et al. used electroporation, introducing DNA for CEA- and HER2-positive colon cancer, and used TLR7 agonists to induce IFNα to enhance the overall efficacy [31]. In the treatment of melanoma, Finocchiaro et al. selected IL-12 and GM-CSF cytokines in order to enhance the immunotherapy effect of suicide gene preparation [32]. Switaj et al. selected CpG ODN 1826 to introduce Th1 immune response, IFN-γ secretion, and B-lymphocyte activation, assisting the proliferation of interleukin-12 in mouse tumor gene therapy [33]. Ryuta Saito used IFN-β gene therapy against malignant gliomas while using DCs stimulated by tumor cell lysates to significantly extend the survival time in mice [34]. These studies have successfully introduced the immune response into gene therapy, improving the antitumor effects. However, the most commonly used approach induces immunostimulation by simply combining immune adjuvant ingredients, making preparation and application inevitably complicated. Therefore, a simple and effective stimulation method is needed. In this study, we focused on the vector itself, modifying the delivery vector DMP with *Lactobacillus reuteri* lysate, thereby endowing it with immunostimulatory functions. According to our cell experiments, DMPLAC markedly increased the expression of DC co-stimulatory molecules, M1 polarization of macrophages, T cell activation, and cytotoxicity after APC presentation. Based on our in vivo experiments, DMPLAC increased immune cell infiltration in tumor tissues, T-cell activation by 30.24%, and the proportion of cytotoxic CD8+ T-cell proportion by 2.93 times. In summary, DMPLAC can shift the TME toward an immunoactivated state, improve antigen presentation, activate T cells, and increase their cytotoxicity. In animal studies, DMPLAC achieved a tumor suppression rate of 29.26% in subcutaneous tumor models. In the abdominal metastasis model, the tumor suppression rate reached 37.57%. The successful preparation of DMPLAC could have been due to the potential of DMP, which provided a core–shell structure that allowed the lysate to be wrapped in. When assembling the nanoparticles, the bacterial lysate was successfully packaged into a cavity to obtain DMPLAC. For the bacterial lysate, the DMP skeleton can enhance the tumor penetration capacity and protect the bioactivity of the lysate, preventing its rapid removal by phagocytes. Studies have shown that *Lactobacillus reuteri* has antitumor bioactivity itself [35], which it exerts by activating various immune cells through peptidoglycan, LPS, lipoteichoic acid, or other components [36]. After the cell wall of *Lactobacillus reuteri* is broken with high-pressure wall-breaking technology to improve safety, fragments can also expose more heterologous antigens and be more easily taken up, making the most of its immunostimulatory properties. Therefore, the *Lactobacillus reuteri* lysate and DMP gene vector used in this study can be described as mutually beneficial.

Immunosuppression reduces the immunogenicity of cancer cells and attenuates immune response sensitivity to help cancer cells resist attack, thereby favoring tumor metastasis and limiting therapeutic benefits. Thus, overcoming the drawbacks of immunosuppression remains a major challenge in immunotherapy to achieve maximum benefits. Using vaccines prepared with antigen ovalbumin and adjuvant CpG, Hu et al. delivered the Spam1 gene expressing hyaluronic acid, degrading the tumor extracellular matrix and sequentially promoting infiltration of immune cells to improve antitumor efficiency [37]. Chen et al. developed a nanoparticle of acid-activatable DOX prodrug and siRNA of PD-L1 to induce apoptosis of tumor cells and reverse immunosuppression [38]. Wang et al. designed a polymeric nanoparticle loading siANRIL to promote apoptosis and prevent proliferation of tumors, and they improved the effect on hepatoma carcinoma by applying TIGIT/PVR inhibitor ^D^TBP-3 peptides [39]. Although these studies all successfully broke immunosuppression to a certain degree, few of them noted that the cooperation of breaking immunosuppression and regulating TME can improve the therapeutic benefit to a new level. The immune checkpoint CD47 was selected as the target spot. By making cancer cells more easily recognized and cleared, siCD47 cooperates with the activation effect of DMPLAC. In vitro experiments proved that DMPLAC could deliver siCD47 efficiently, knocking out the CD47 gene in CT26 cells by 45% and reducing the CD47 protein on the surface by 34.13%. In the peritoneal metastasis model, the number of immune cells in the tumor microenvironment of the DMPLAC/siCD47 group was significantly increased. The activation of T cells and M1 macrophages increased by 31.65% and 16.97%, respectively, based on the DMPLAC regulation. In the animal experiments, the addition of siCD47 further inhibited tumor progression by 42.87% on basis of DMPLAC in the intraperitoneal metastasis model and by 15.61% in the subcutaneous tumor model. This combination of immune activation and immunosuppression breaking improved the overall therapeutic effect; therefore, we speculate that the superior antitumor effect of co-loaded nanocomplexes may be caused by the cooperation of regulating TME and reversing immunosuppression. In the DMPLAC/siD47 complex, bacterial lysate–modified DMPLAC acts as an immunostimulant, whereas siCD47 blocks the SIRP-α/CD47 pathway to improve the immunogenicity of tumor cells, making it easier for them to be cleared by immune cells activated by DMPLAC. The effects on immune cells and tumor cells can coordinate with each other.

In the final analysis, all these benefits were attributed to the unique structure of DMP, which is an efficient carrier that can load stimulatory molecules and deliver genetic material. On the one hand, it is self-assembled by the cationic liposome DOTAP and amphiphilic block copolymer mPEG-mPCL, which forms a cavity structure and allows the bacterial lysate to enter. However, the positive charge is completely unaffected by the internal cargo, loading nucleic acid by electrostatic adsorption, thereby providing carrier support for the DMPLAC/siCD47 complex to achieve dual function. In fact, some progress has been made in studies of cationic nanomaterials in recent years [40]. However, current research focusing on gene delivery is usually limited to improving delivery capacity, and it is difficult to find suitable vectors to achieve co-delivery of genes and immune stimulants. The multifunctional cationic carrier DMP prepared has successfully achieved efficient simultaneous delivery of bacterial lysate and siCD47, and has shown satisfactory results in various experiments, both in vitro and in vivo.

## 5. Conclusions

Herein, we prepared a novel siRNA delivery vector, DMPLAC, based on intestinal probiotic lysates that can simultaneously activate the immune response at the same time. Loaded with siRNA targeting CD47, the DMPLAC/siCD47 complex can achieve dual immune modulation. A variety of in vitro and in vivo immunostimulation experiments, a mouse abdominal tumor model, and a subcutaneous tumor model also verified the satisfactory therapeutic effect of the complex. The experimental results suggest that this is a potential strategy for cancer immunotherapy.

## Data Availability

The raw data supporting the conclusions of this article will be made available by the authors on request.

## Supplementary Materials

The following supporting information can be downloaded at: https://www.mdpi.com/article/10.3390/pharmaceutics17020139/s1, Figure S1: The gating strategies of the study about immunostimulation of DMPLAC in vitro. Figure S2: The gating strategies of the study about immunostimulation of DMPLAC in vivo of Figure 4. Figure S3: The gating strategies of the study about immunostimulation of DMPLAC in vivo of Figure 5. Figure S4: The gating strategies of transfecting in vitro and Endocytosis and Trafficking Mechanism Analysis. Figure S5: The gating strategies of the study of expression of CD47 on CT26. Figure S6: The gating strategies of the study about TME of mice in abdominal metastasis model of Figure 7. Figure S7: Blood related safety analysis of the DMLAC/siCD47 complex (n = 3). Figure S8: DMPLAC demonstrated tumor inhibition in the subcutaneous model of CT26. (A) Experimental design diagram. (B) The average tumor growth curves in all treatment groups (** *p* < 0.01, **** *p* < 0.0001) (n = 5). (C) Changes of body weight in the NS group and each treatment group (n = 5). (D) Tumor Images collected from each mouse in all treatment groups (n = 5). (E) The average number of tumor nodules collected in the NS group and each treatment group (** *p* < 0.01, *** *p* < 0.001) (n = 5). (F) Tumor inhibition rate in the NS group and each treatment group (* *p* < 0.05, ** *p* < 0.01).
