# Supplementary material for: Intestinal Probiotic Lysate Modified Bifunctional Nanoparticle for Efficient Colon Cancer Immunotherapy
Source: Pharmaceutics. 2025 Jan 21;17(2):139. doi: 10.3390/pharmaceutics17020139 (PMC11859493; doi:10.3390/pharmaceutics17020139)
Supplement: Supplementary file 1 [file pharmaceutics-17-00139-s001.zip › pharmaceutics-3370219-supplementary.pdf]

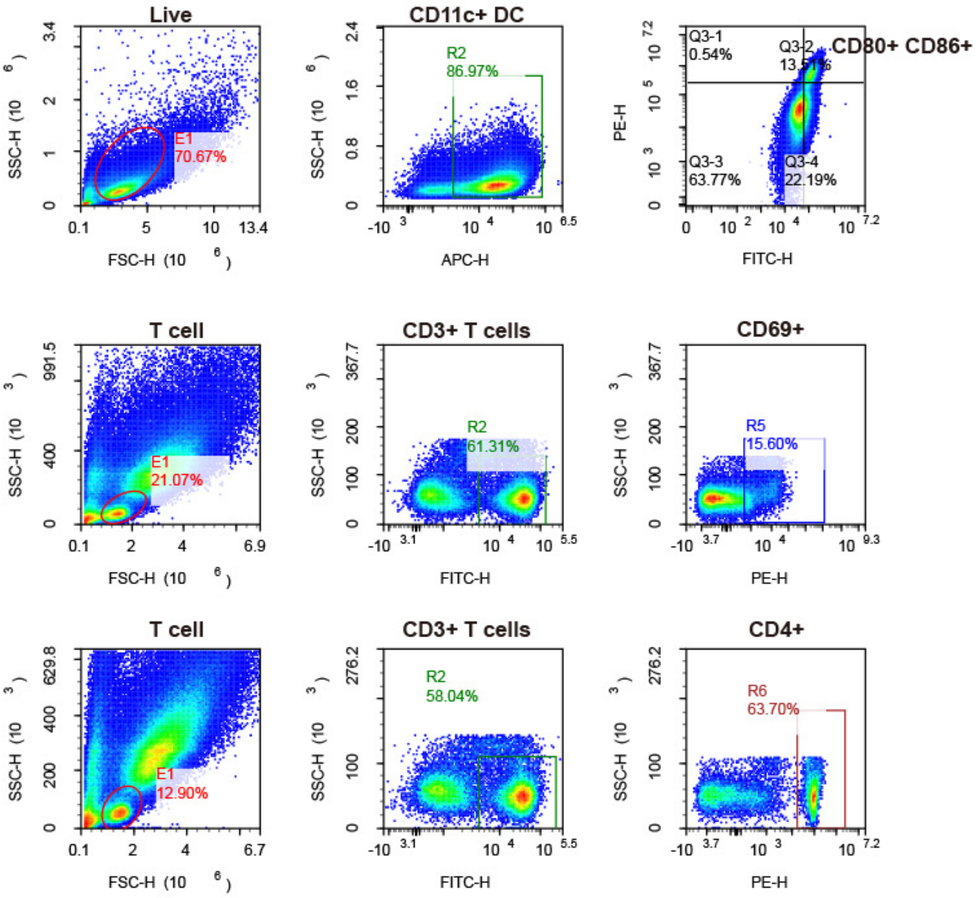

Figure S1: The gating strategies of the study about immunostimulation of DMPLAC in vitro.

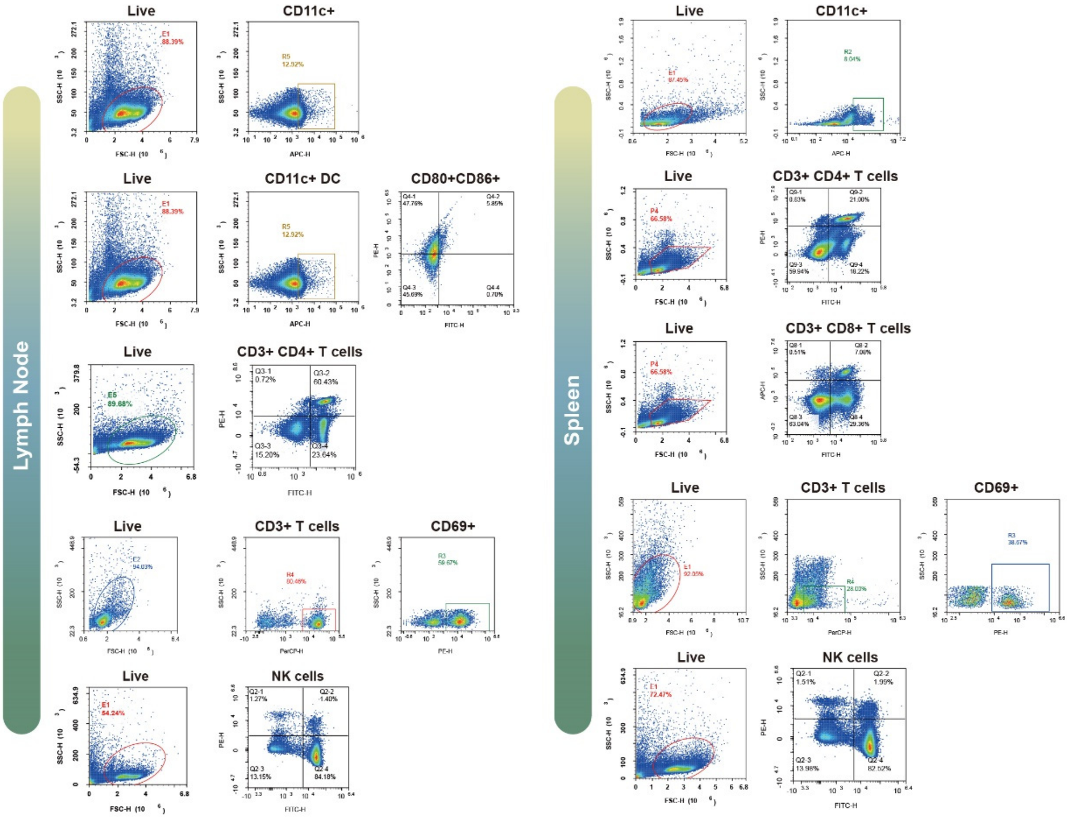

Figure S2: The gating strategies of the study about immunostimulation of DMPLAC in vivo of Figure 4.

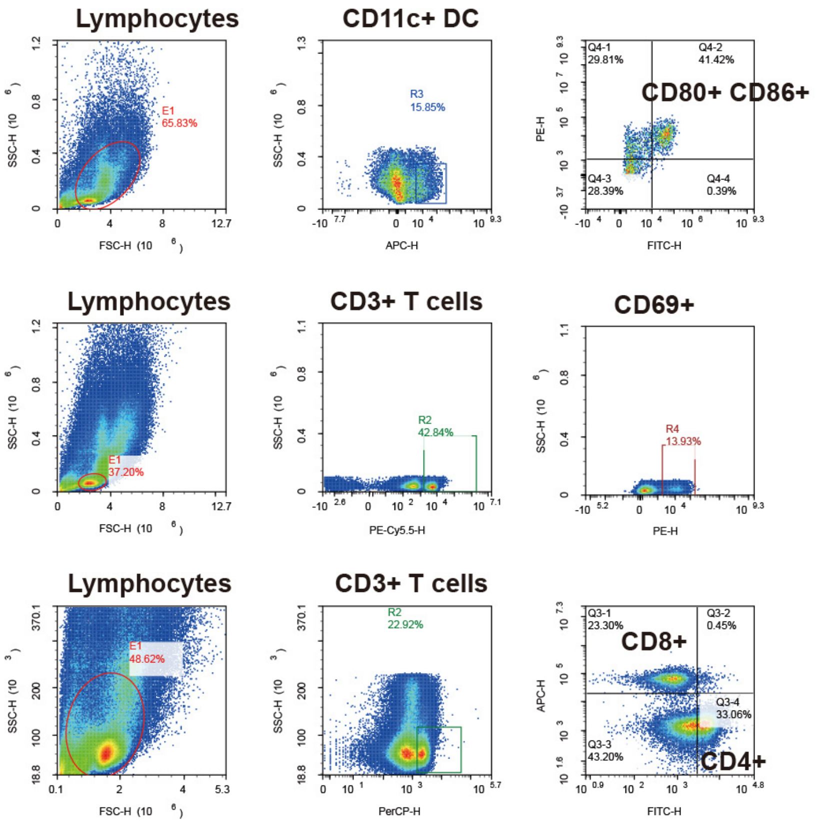

Figure S3: The gating strategies of the study about immunostimulation of DMPLAC in vivo of Figure 5.

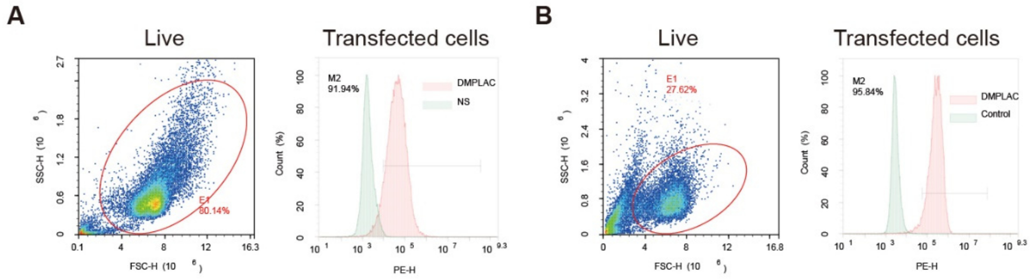

Figure S4: The gating strategies of transfecting *in vitro* and Endocytosis and Trafficking Mechanism Analysis.

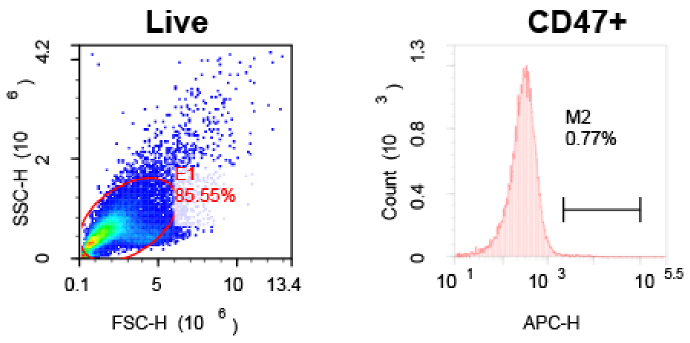

Figure S5: The gating strategies of the study of expression of CD47 on CT26.

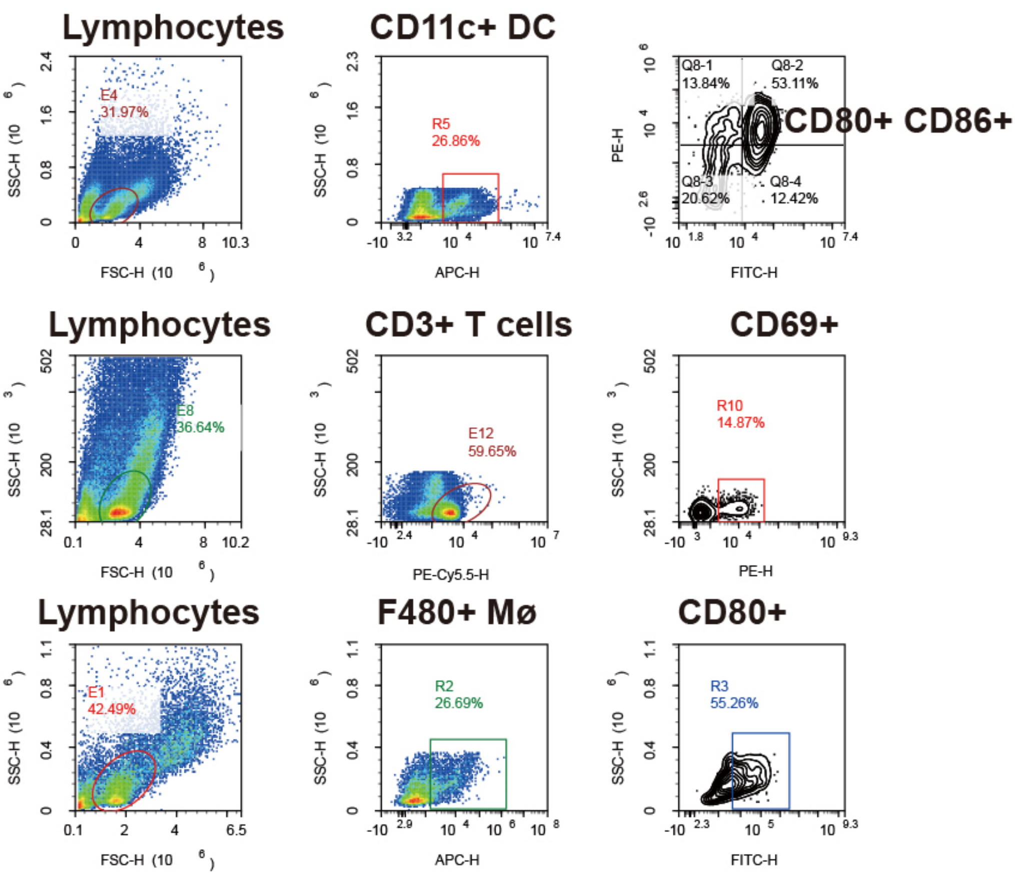

Figure S6: The gating strategies of the study about TME of mice in abdominal metastasis model of Figure 7.

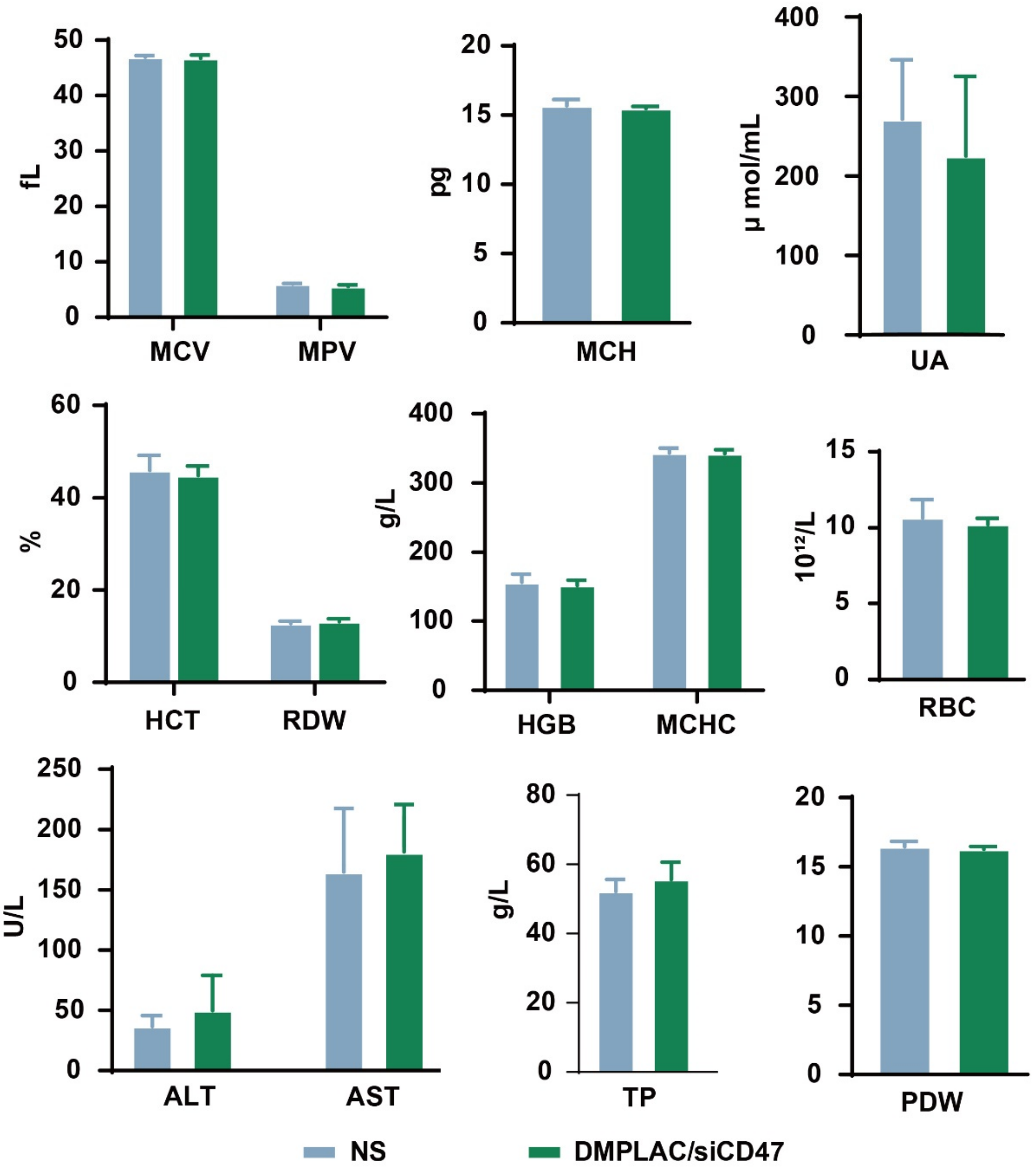

Figure S7: Blood related safety analysis of the DMLAC/siCD47 complex (n=3).

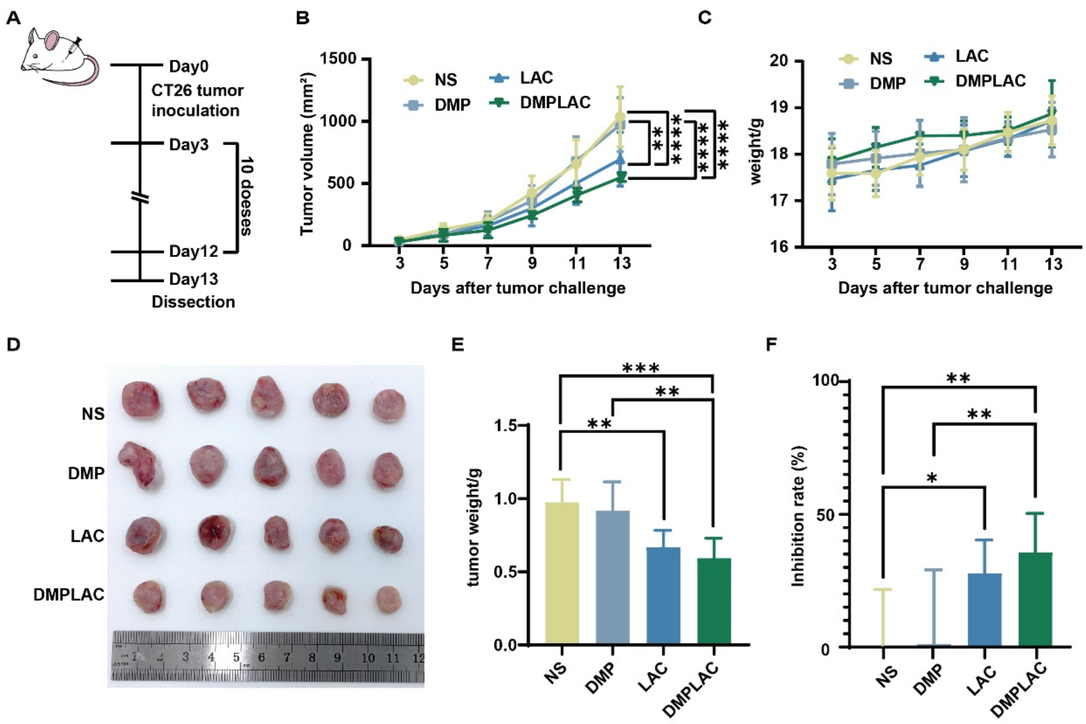

Figure S8: DMPLAC demonstrated tumor inhibition in the subcutaneous model of CT26. (A) Experimental design diagram. (B) The average tumor growth curves in all treatment groups (**p<0.01, ****p<0.0001) (n=5). (C) Changes of body weight in the NS group and each treatment group (n=5). (D) Tumor Images collected from each mouse in all treatment groups (n=5). (E) The average number of tumor nodules collected in the NS group and each treatment group (**p<0.01, ***p<0.001) (n=5). (F) Tumor inhibition rate in the NS group and each treatment group (*p<0.05, **p<0.01).
